# Combined visual and motor disorganization in patients with schizophrenia

**DOI:** 10.3389/fpsyg.2013.00620

**Published:** 2013-09-18

**Authors:** Anne Giersch, Hélène Wilquin, Rémi L. Capa, Yvonne N. Delevoye-Turrell

**Affiliations:** ^1^Department of Psychiatry, INSERM U1114, University Hospital of StrasbourgStrasbourg, France; ^2^LPCLS EA3278, Aix Marseille UniversityAix-en-Provence, France; ^3^URECA, University Lille Nord de FranceLille, France; ^4^CNRS, MESHS, USR 3185Lille, France

**Keywords:** schizophrenia, motor control, disorganization symptoms, visual organization, visual perception, visual grouping

## Abstract

Cognitive impairments are difficult to relate to clinical symptoms in schizophrenia, partly due to insufficient knowledge on how cognitive impairments interact with one another. Here, we devised a new sequential pointing task requiring both visual organization and motor sequencing. Six circles were presented simultaneously on a touch screen around a fixation point. Participants pointed with the finger each circle one after the other, in synchrony with auditory tones. We used an alternating rhythmic 300/600 ms pattern so that participants performed pairs of taps separated by short intervals of 300 ms. Visual organization was manipulated by using line-segments that grouped the circles two by two, yielding three pairs of connected circles, and three pairs of unconnected circles that belonged to different pairs. This led to three experimental conditions. In the “congruent condition,” the pairs of taps had to be executed on circles grouped by connecters. In the “non congruent condition,” they were to be executed on the unconnected circles that belonged to different pairs. In a neutral condition, there were no connecters. Twenty two patients with schizophrenia with mild symptoms and 22 control participants performed a series of 30 taps in each condition. Tap pairs were counted as errors when the produced rhythm was inverted (expected rhythm 600/300 = 2; inversed rhythm <1). Error rates in patients with a high level of clinical disorganization were significantly higher in the non-congruent condition than in the two other conditions, contrary to controls and the remaining patients. The tap-tone asynchrony increased in the presence of connecters in both patient groups, but not in the controls. Patients appeared not to integrate the visual organization during the planning phase of action, leading to a large difficulty during motor execution, especially in those patients revealing difficulties in visual organization. Visual motor tapping tasks may help detect those subgroups of patients.

## Introduction

Many studies have shown that patients with schizophrenia have a difficulty organizing visual information through space (Place and Gilmore, 1980; Wells and Leventhal, 1984; Silverstein et al., 2000; Must et al., 2004; Uhlhaas et al., 2006; Kurylo et al., 2007; Giersch and Rhein, 2008; van Assche and Giersch, 2011). These deficits are often correlated to the clinical symptom of disorganization, and might even reflect this symptom [review in Silverstein and Keane (2011)]. The possibility to objectify this clinical symptom is of importance since it is related to the loosening of associations that has been proposed as a core feature in schizophrenia (Bleuler, 1911). However, cognitive impairments observed in stabilized patients are of relatively small amplitude. Contrary to brain-damaged patients, the results in stabilized patients with schizophrenia do not lead to all-or-none phenomena, making it difficult to relate cognitive impairments and clinical symptoms, or to derive subgroups of patients according to their cognitive difficulties. Yet, some cognitive deficits might be specific to schizophrenia (Loughland et al., 2002; Lee et al., 2013), and they are often correlated to clinical symptoms (Silverstein and Keane, 2011). We reasoned that neuropsychological explorations may need to be further adapted to the specific difficulties encountered in schizophrenia. Second, a one-to-one correspondence is unlikely to exist between cognitive disorders and clinical symptoms. Cognitive disorders more likely interact with one another. Here, we illustrate this idea by elaborating a new test based on known impairments that might characterize schizophrenia and some of its associated symptoms. We devised a task that requires both (1) the perceptual organization of information, and (2) the motor organization of sequences of action plans through time and space. Our aim was to assess how the impairments in these fields conjointly contribute to the difficulties observed in patients, and whether the visuo-motor coordination constraints imposed by the task would lead to larger impairments than usual in patients with schizophrenia displaying mild symptoms, specifically those characterized by the clinical symptom of disorganization.

### Organization of a motor sequence

We have shown previously that patients with schizophrenia display difficulties in organizing and smoothly executing motor actions when those actions are sequential (Delevoye-Turrell et al., 2007). Even when performing a task as simple as lifting and gripping an object, our previous results suggest that patients with schizophrenia fail to plan their actions in advance. Contrary to controls, they are not disturbed by a secondary task during the planning phase, but use more resources than healthy volunteers during the motor execution phase of movements (Delevoye-Turrell et al., 2006). Other studies have also reported a difficulty in planning actions in a coherent way (Jogems-Kosterman et al., 2001; Zalla et al., 2006; Grootens et al., 2009). However, to the best of our knowledge, previous studies have not shown any correlation between clinical disorganization and the ability to plan sequences of motor actions. In the present study, we included a manipulation of those visual factors that are known to be affected by clinical disorganization in order to increase the sensitivity of our task to this clinical factor.

### Organization in visual perception

Mechanisms helping to organize visual information are diverse, and intervene at different stages of visual organization. Visual information is first extracted locally and decomposed into color, orientation, or luminance information. This information then needs to be correctly integrated or separated in order to derive the shapes of objects and the structure of the considered visual scene. Many automatic grouping mechanisms have been described, e.g., grouping by color, by similarity, by uniform connectedness (Wertheimer, 1923/1950; Palmer and Rock, 1994). These grouping mechanisms allow us to attribute object parts to the same object and to put together different objects that belong to a same group, e.g., the trees in a forest. Our results and those reported in the literature show that mildly symptomatic patients with schizophrenia benefit as much as controls from automatic grouping (Chey and Holzman, 1997; Silverstein et al., 1998; Giersch and Rhein, 2008; van Assche and Giersch, 2011), at least when grouping cues are unambiguous and when they do not lead to spurious grouping (see Kurylo et al., 2007; Silverstein and Keane, 2011 for limits to the benefits of automatic grouping in schizophrenia).

In addition to automatic grouping mechanisms, more integrated cognitive mechanisms provide a function of “re-grouping” of visual elements (van Assche and Giersch, 2011) This mechanism would give the means to the brain to explore a visual scene by foveating different objects successively, without being restrained by the fact that these objects belong to different groups of objects. We have suggested that patients with schizophrenia are in great difficulty when having to “re-group” items in visual perceptual tasks, and shown that this effect is correlated with clinical disorganization (van Assche and Giersch, 2011). To further assess the possible consequences of regrouping deficits in schizophrenia, here we question the possibility that “re-grouping” is also involved in the planning and execution of a sequence of motor actions that requires participants to point toward objects of different groups. Indeed, when pointing to a series of visual targets, these targets can be considered individually (i.e., one by one) or as a series of sub-elements of a greater whole (two adjacent targets composing a group and guiding sequential pointing actions). The grouping of adjacent targets might then affect the planning and fluent execution of the motor sequences.

The possibility that visual information can impact action planning and execution is supported by several arguments, which are presented in the next section.

### Visual perception and action

A growing number of empirical findings now support the involvement of perception in action processing, and has led to the ideomotor theory (Prinz, 1987; see Nattkemper et al., 2010; Shin et al., 2010, for reviews). According to this theory, the brain would use perceptual representations of action-effects for optimal planning of motor actions. For example, if one plans to manually point toward visual targets, the sensory feedback expected to result from this action is processed in advance of action initiation, i.e., during motor planning. During action execution, participants can then check whether the actual sensory feedback corresponds to what was expected. As a consequence, the correspondence between the action and sensory feedback has a facilitation effect on action, as shown by a number of studies [review in Shin et al. (2010)]. For example, Kunde (2001) used a task during which key presses were followed by tones of varying intensity. The results showed that the correspondence between the intensity of the key presses and the loudness of the tones facilitated the responses. Even when the relationship between the action and its consequence is less straightforward, it can be learned and used to optimize performance (Elsner and Hommel, 2004). In the present study, participants had to execute pairs of pointing actions toward distinct visual targets, and we manipulated the correspondence between the organization of the motor sequence on the one hand and the perceptual organization of visual targets on the other hand. The manipulation of this correspondence was then used in the framework of the ideomotor theory to assess to what extent patients with schizophrenia are able to include visual representations for motor planning and to what extent their difficulties in organizing perceptual information may be revealed in a task that requires in addition the execution of sequences of motor actions.

### Paradigm and predictions

**Figure 1:**
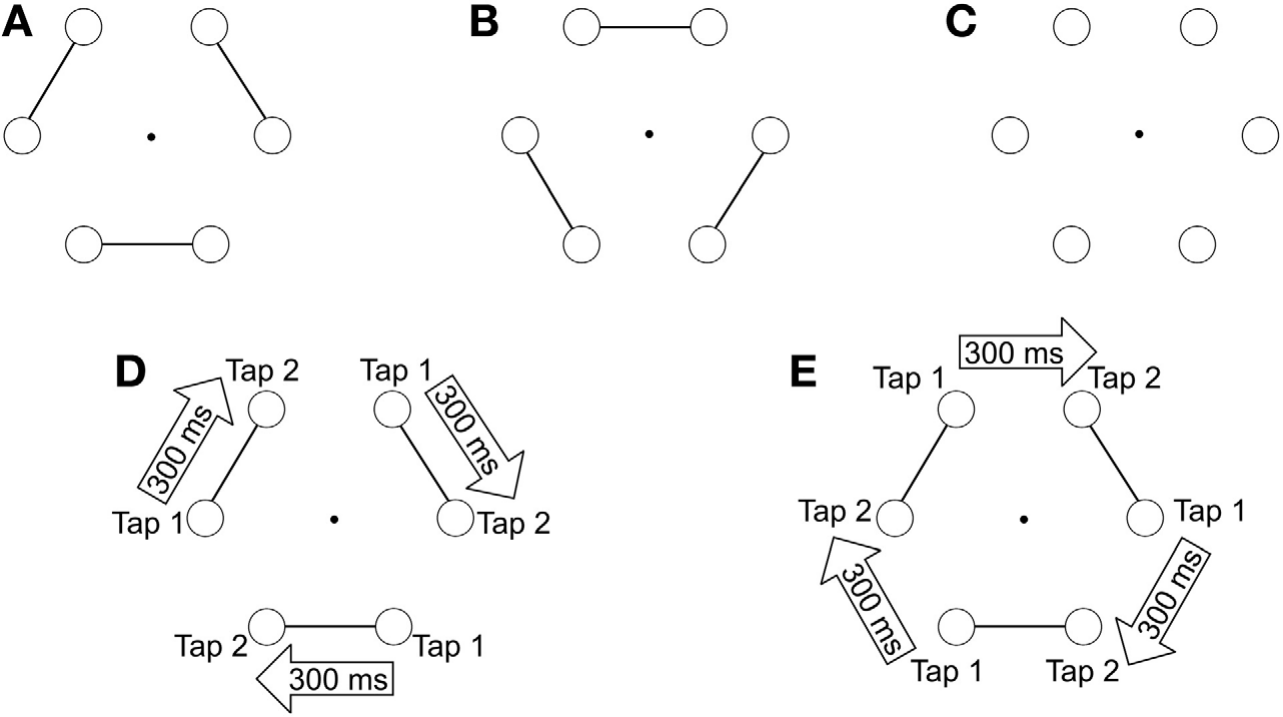
**(A,B)** present the two images used to test the influence of visual grouping (connecters) on motor performances. **(C)** was used in the neutral condition (absence of connecters). **(D,E)** illustrate the tapping task on image A, during which participants are required to execute pairs of taps on adjacent circles. The two taps of a pair are separated by short intervals of 300 ms, whereas pairs are separated from one another by longer intervals of 600 ms. In the congruent condition, pairs of taps are to be executed on connected circles **(D)**, whereas in the non-congruent condition, pairs of taps are directed toward unconnected circles **(E)**.

In the present study, we describe a manual-pointing task in which participants were required to tap on 6 circles (the visual targets) that were presented on a tactile screen (Figure 1). The participants' task was to tap successively and clockwise on the targets following the rhythm of a series of auditory stimuli. To induce the perception of an auditory structure, we used temporal proximity, which is known as a fundamental principle of organization in auditory perception (Wertheimer, 1938). Two sounds separated by a short interval defined a pair, and successive pairs were separated by a longer interval. Since participants were instructed to tap in synchrony with the sounds, the rhythmic organization of the auditory stimuli induced a rhythmic motor execution of pairs of taps on adjacent circles, which required participants to structure the motor elements through space and through time. When participants performed the pairs of taps on 6 isolated circles (without any visual grouping cues), the motor outputs were organized in pairs but the perceptual representation of the visual targets was not. A perceptual visual structure was induced by using connectors, which linked adjacent circles together in order to create visual perceptual pairs. This visual structure globally matched the auditory structure, inasmuch both organizations led to the perception of pairs: sound pairs on the one hand, and visual pairs on the other hand. Consequently, the perceptual organization of the circles could hypothetically affect the motor output, depending on the grouping abilities of the participants. Indeed, when pairs of taps were executed on pairs of connected targets the action structure matched the automatic visual organization. On the other hand, when the pairs of taps were to be executed on unconnected targets, participants had to match their pair of taps with “re-grouped” targets (Figure 1). We hypothesized that patients would show impairments related to their difficulties in visual “re-grouping,” i.e., we expected patients to have difficulties when the task required to point to successive visual targets belonging to different groups. Inasmuch the difficulty related to “re-grouping” is related to clinical disorganization, we also predicted that this pattern of results should be observed predominantly in those patients suffering from clinical disorganization.

## Methods

### Participants

Twenty-two stabilized outpatients participated in the study. They met the Diagnostic and Statistical Manual of Mental Disorders (Fourth Edition)'s criteria for schizophrenia (American Psychiatric Association, 1994). The diagnosis was based on a semi-structured interview (the Mini International Neuropsychiatric Interview, Sheehan et al., 1998), and was established by a senior psychiatrist of the University Psychiatry Department. Patients were 9 women and 13 men (mean age = 40.2 years, *SD* = 8.2; schooling years = 11.5, *SD* = 2.4).

The patients' performances were compared to those obtained in 22 controls (11 women and 11 men; mean age = 40.9 years, *SD* = 7.7; schooling years = 13.2, *SD* = 2). The two groups did not differ in age (*F* < 1). They did differ, however, in the number of schooling years [*F*_(1, 42)_ = 6.2, *p* < 0.05).

Regarding clinical characteristics, mean disease duration was 14.9 years (*SD* = 7.6). Symptoms were assessed through the use of the Positive and Negative Syndrome Scale (PANSS, Kay et al., 1987). Patients presented a mean score of 16.8 (*SD* = 5.9) for the Positive Subscale, 22.1 (*SD* = 6.3) for the Negative Subscale and 36.5 (*SD* = 9.8) for the Global Psychopathology Subscale, leading to a total mean score of 75.5 (*SD* = 17.2). Overall, these results indicated that the patients were within the normal-to-mild range, and were relatively asymptomatic.

Individual PANSS scores were also converted into a Positive factor (sum of P1, P3, G9 = 8.2, *SD* = 3.5), a Negative factor (sum of N1, N2, N3, N4, N5, N6, N7, G7, G13, and G16 = 20.3, *SD* = 6.3) and a Disorganization factor (sum of P2, N5, G10, and G11 = 9.6, *SD* = 3.1), according to the classification proposed by Lépine et al. (1989).

We were especially interested in the disorganization factor, and thus, we divided patients in two subgroups, one with a disorganization score above or equal to 10 (*N* = 10) and another group with a disorganization score below 10 (*N* = 12). This cutoff was set in order to divide the group of patients in two (this could not be totally achieved, due to the fact that 4 patients had a disorganization score of 9). These two groups differed in the number of schooling years [10.4 *SD* 1.6 for the group with a high disorganization score vs. 12.5 *SD* 2.5 for the group with a low disorganization score, *F*_(1, 20)_ = 5.1, *p* < 0.05]. We thus, divided the group of controls according to their schooling years also, in order to evaluate whether there was an impact of schooling years on the performance patterns. The 10 controls with the lowest number of schooling years had a similar education level as the patients from the group with a high disorganization score [10.4 in patients vs. 11.2 in controls, *F*_(1, 18)_ = 2.2, *n.s*.]. It is to note that the two subgroups of controls revealed very similar performances profiles, indicating that the education level did not impact result patterns. Our findings were in fact similar whether we divided the group of controls or not. Thus, for the sake of simplicity, the data presented in the following section corresponds to the means averaged across the two subgroups of controls.

All patients were taking medication, and doses were converted in chlorpromazine equivalents (mean dose = 272 mg, *SD* = 237). Patients received a neuroleptic treatment, either typical (*N* = 7) or atypical (*N* = 15). Six patients were also administered with an antiparkinsonian treatment.

All participants gave written informed consent prior the beginning of the study, consistently with the Declaration of Helsinki's recommendations. This project was approved by the local ethics committee (Comité Consultatif de Protection des Personnes dans la Recherche Biomédicale d'Alsace IV). Any history of neurological disorder (meningitis, brain injury, cerebrovascular accident), generalized anaesthesia within the past 3 months, or recent drug abuse, was considered as exclusion factors. Participants treated by benzodiazepines were also discarded from this study. In addition, four participants were excluded from the initial group of 24 participants because they did not follow instructions (2 patients and 2 controls).

### Equipment and stimuli

Participants were seated comfortably on a chair in front of a touch screen (Elo Touch, 23 × 36 × 30 cm), which was placed on a narrow support at knee-height. The participants' task was to produce sequential-pointing movements to visual targets, with their dominant index finger, in synchrony with a series of beeps produced by the computer. The visual targets consisted in 6 equidistant outlined black circles arranged in the form of a hexagon. Participants were asked to point each circle (diameter 1.2°), one after the other, starting with the bottom-right circle (Figure 1). Participants were required to point each target (touching the screen) following a clockwise direction until they had reached the end of the trial duration (13 s). The auditory rhythmic sequence of beeps was created using Audacity software. Three different visual images were created using CorelDraw software. One was with the 6 circles but without connecters. The two others were with connecters, i.e., with line-segments linking neighboring circles by pairs (Figure 1). There were two possible locations for the connecters and both were used an equivalent number of times. Participants were instructed to be as accurate as possible both spatially and temporally. The connecters being presented right from the start of each trial, they emphasized a first grouping structure to the task, based on the perceptual principles of visual grouping.

### Experimental conditions

The participants' task was to tap each visual target in synchrony with the sounds emitted by the computer. Alternating rhythmic patterns were used, i.e., inter-stimulus intervals of 300 and 600 ms. These alternating intervals imposed an auditory grouping and emphasized the need to re-group motor actions. Sounds separated by a short interval (300 ms in our paradigm) induce the perception of grouped pairs of sounds because they are close in time and separated from other pairs by a longer interval (600 ms in our paradigm). Since participants were required to tap in synchrony with the sounds, this led to the execution of pairs of taps close together in time, the two taps of a pair being ideally separated by a time interval of 300 ms, and successive pairs of taps being ideally separated by a time interval of 600 ms. The 300/600 ms intervals were chosen because when combined they are close to the preferred tempo of Inter Response Intervals of 500 ms which corresponds to the spontaneous tempo reported in the tapping literature (Repp, 2005). In addition, we knew from previous studies (Ameller et al., 2011; Turgeon et al., 2012) that patients with schizophrenia are able to perform the task at this speed. Finally, the contrast between 300 and 600 ms intervals was strong enough to yield the unambiguous perception of rhythmic grouping. It should be noted that this auditory grouping manipulation has already been applied to taps executed in one unique location; the originality of our paradigm is to ask participants to tap on distinct visual targets spread out throughout the action space and thus, requiring participants to re-group motor elements not only through time but also through space.

The 300/600 ms rhythmic pattern was used both with stimuli without and with connecters, leading to three conditions:

1. *A neutral condition:* Participants were required to tap alternating rhythmic patterns on visual targets that were not connected.
2. *A Congruent condition:* Connectors were used to group visual targets together two by two. The rhythmic structure of the auditory beeps led participants to execute pairs of taps (inter-stimulus interval 300 ms) on connected visual targets. Long inter-stimulus intervals of 600 ms separated the pairs of taps and corresponded to unconnected visual targets (see Figure 1D).
3. *A Non-Congruent condition:* This condition was the complementary situation to the congruent condition. Connectors were used to group visual targets, but this time the rhythmic structure of the auditory beeps led participants to execute pairs of taps (inter-stimulus interval 300 ms) on unconnected visual targets, i.e., the targets belonging to different pairs. Long inter-stimulus intervals of 600 ms separated the unpaired taps and corresponded to connected visual targets (see Figure 1E).

### Experimental design

The experiment lasted about 1 h. First, all participants performed a familiarisation phase in which the different rhythms and images were presented. Participants were trained to synchronize their pointing taps with a trial requiring no visual grouping and equivalent time intervals between tones before being trained with alternating rhythms.

Each trial consisted in a ***listening phase*** (≈4.5 s), a ***waiting phase*** without sound, which gave time for the participants to get ready to point to the first circle [they prepared their finger just above the first circle (bottom right)] (≈3 s) and a ***test phase*** of 30 taps (≈13 s). Each participant performed a sum-total of 48 trials.

Participants started with the Neutral condition (without connecters) and performed 2 blocks of 4 trials each. This was followed by the Visual Grouping conditions. Each congruent and non-congruent condition included 4 blocks of 5 trials each (2 blocks with the image A and 2 with the image B, see Figure 1). These 8 blocks were randomized.

### Statistical analysis

For each subject and each trial, we measured both the Inter Response Interval and the asynchrony between the tap and the sound. The spatial location of the tap was also measured allowing us to distinguish those taps directed toward connected and unconnected targets. We were in this way able to check whether participants followed the rhythm correctly.

In a first analysis, we measured the Inter Response Interval (IRI in ms), which is the time interval between the onsets of two successive taps produced by the participants, and which is a parameter that is commonly used in the tapping literature (Repp, 2005). From this IRI measure, we then calculated the mean ratio across trials for each participant and under each condition. This ratio referred to the participants' capacity to maintain the metric organization of the auditory rhythmic sequence (i.e., alternance of short and long intervals lasting 300 and 600 ms, respectively) and was calculated following the equation: IRIn/IRIn-1, where n refers to the long interval (600 ms), and n-1 to the short interval (300 ms). The ratio should thus be equal to 2. When this ratio was inverted (<1), we considered the trial as bad. Following this rule, we then calculated for each subject and under each experimental condition, the percentage of trials considered as bad (the number of bad trials divided by the total number of trials in a condition), a calculation that we will refer to in the following as “rate of errors.”

In a second analysis we aimed to evaluate the use of anticipatory mechanisms. We used here another typical parameter that is used in tapping experiments, i.e., the tap-tone synchronization accuracy (applied on correct trials only). To that aim, we calculated the asynchrony, which was taken as the time interval between the peak force of each tap and the start of the nearest tone (in ms).

A crucial aspect of such a sensorimotor synchronization task is the predictability of the external beep, which arises from its regular recurrence. It is this predictability that allows good synchronization between tap and sound. This is specifically the feature that distinguishes sensorimotor synchronization from a simple reaction time task, for which the response is made as quickly as possible after the sound and thus, is characterized by a delay >180 ms. In rhythmic tapping, the ability to anticipate the beep occurrence leads to asynchronies between tap and sound that are close to zero or even negative in healthy volunteers. Tap-tone asynchronies can thus be used to evaluate the quality of predictive timing. In addition, in the present study, our task imposed an organization of the taps by pairs, and this means that predictive timing might differ according to the order of the taps. We thus, distinguished the tap-tone asynchronies for the 1st and 2nd tap of a pair.

We used Analyses of Variance (ANOVA) with repeated measures on both error rates (rates of trials with an inverted ratio) and tap-sound asynchronies, with Group as a between-group factor and Experimental Condition as a within group factor (neutral, congruent and non-congruent conditions). For tap-sound asynchronies, an additional within-group factor was the tap Order within the sequences of two taps (first vs. second). Significant interactions were decomposed by means of Tukey HSD *post-hoc* tests and Sub-Analyses of Variance.

## Results

### Error rates

The most striking effect was the large amount of errors characterizing motor performances in the patients with schizophrenia. The interval ratio of patients was inverted, i.e., below 1, in 18% of the trials (averaged across conditions), which was significantly higher than the rate of inversions of 5% observed in the controls [*F*_(1, 42)_ = 11.1, *p* < 0.005].

**Figure 2:**
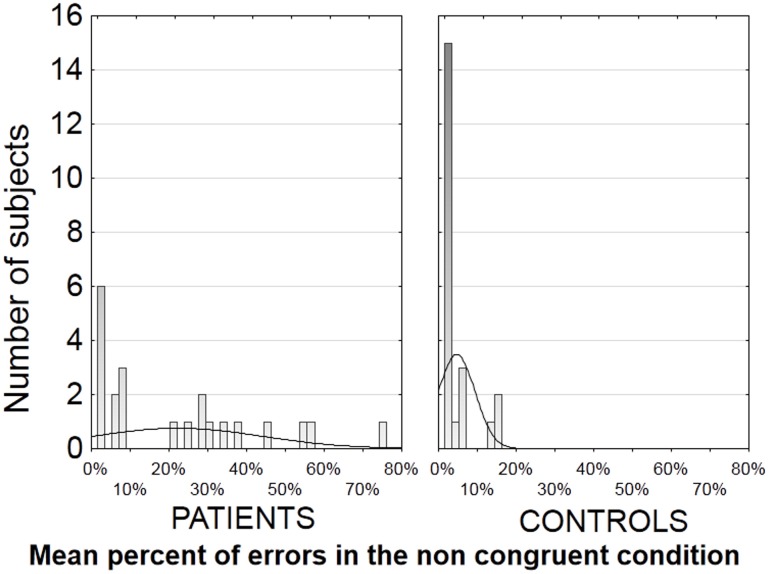
**Distribution of the error rates in the non-congruent condition in each group, i.e., number of participants per error rate, in patients on the left and in controls on the right**. As can be seen on the left panel, 11 patients have an error rate above 19%, which is more than 3 *SD* above the mean error rate observed in the controls.

The error rates were highly variable in patients, but were unusually large in half of the patients in the non-congruent condition. In this condition, 11 patients (vs. 0 control) revealed an error rate above 19%, i.e., 3*SD* above that measured in the controls (4.7% *SD* 4.7) (see distribution in Figure 2). The other half of the patients (*N* = 11) showed an error rate below 10% which was within the normal range (Figure 2). The two subgroups of patients differed significantly only on the score of clinical disorganization [*F*_(1, 20)_ = 4.7, *p* < 0.05]. These results were confirmed by the presence of significant correlations between tapping performances and clinical scores in the patients group as a whole. More specifically, a significant positive correlation was revealed between the score of clinical disorganization and the rate of errors in the non-congruent condition (*r* = 0.44, *N* = 22, *p* < 0.05). There were no other significant correlations with clinical scores, neither with the sub-scales of the PANSS nor with the positive or negative Lepine factors. All further analyses on motor performance scores were hence conducted taking into account the disorganization subgroups.

It is to be noted that when the group of patients was not divided, there was only a tendency toward a significant interaction between groups and experimental conditions [*F*_(2, 84)_ = 2.6, *p* = 0.076], which might be due to the heterogeneity of performance in the patients. Nevertheless, the Tukey HSD *post-hoc* analysis showed that in patients errors were more frequent in the non-congruent (22.1%) than in the congruent condition (14.8%), *p* < 0.05. This effect was not observed in controls (4.7 vs. 3.0%, *p* > 0.9).

In the following, we compared the performance levels across three groups of individuals (controls; patients with a high disorganization score; patients with a low disorganization score) as a function of the experimental conditions.

**Figure 3:**
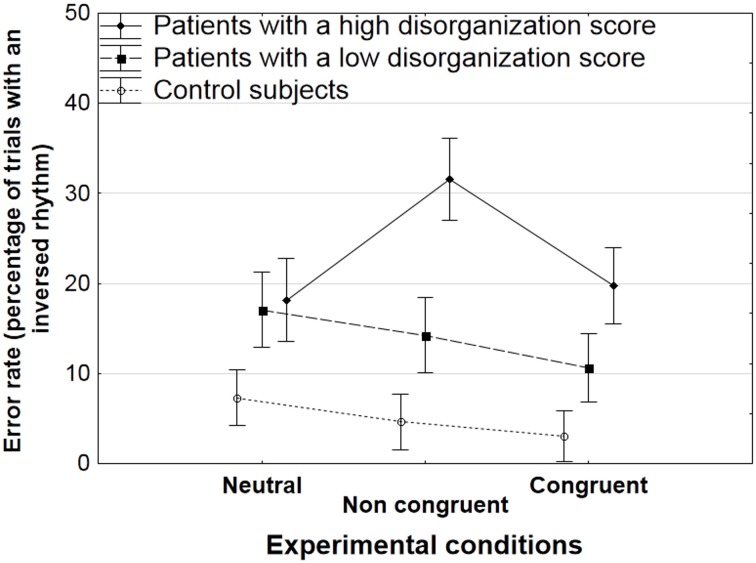
**Mean rate of errors (trials with an inverted rhythm) with standard errors of the means (SEM) as a function of the experimental condition (neutral, non-congruent, congruent) in patients with a high and low disorganization score, and in controls**.

There was a significant interaction between groups and experimental conditions for the rate of errors [*F*_(4, 82)_ = 4.8, *p* < 0.005]. The HSD Tukey *post-hoc* analysis showed that patients with a high score of disorganization had a higher error rate in the non-congruent condition (31.6%) than in the two other conditions (18.2% in the neutral condition, *p* < 0.005; 19.7% in the congruent visual grouping condition, *p* < 0.05, Figure 3). These results were confirmed by means of sub-analyses that were conducted in each group and aimed at comparing performance levels across conditions. The sub-analyses not only confirmed the HSD Tukey *post-hoc* test but also revealed some effects in controls and in patients with a low disorganization score. The effect of experimental conditions was significant in the group with a low disorganization score [*F*_(2, 22)_ = 3.7, *p* < 0.05], due to a lower error rate in the congruent than in the neutral condition (10.6 vs. 17.1%, *p* < 0.05 in the HSD Tukey *post-hoc* analysis following the sub-analysis in patients with a low disorganization score). In the control group, the effect of experimental conditions tended toward significance only, *F*_(2, 42)_ = 3, *p* = 0.06. As in the group with a low disorganization score, the error rate tended to be lower in the congruent than in the neutral condition (3 vs. 7.3%, *p* = 0.05).

Sub-analyses were also used to compare performance levels across groups of participants in each experimental condition. In the non-congruent condition, the group effect was significant [*F*_(2, 41)_ = 11.8, *p* < 0.001]. The HSD *post-hoc* analysis showed that patients with a high disorganization score made more errors than both controls (*p* < 0.001) and patients with a low disorganization score (*p* < 0.05). These results consistently confirm the performance decrement in the non-congruent condition in disorganized patients relative to the two other groups.

In the congruent condition, the group effect was significant [*F*_(2, 41)_ = 5.6, *p* < 0.01] and the HSD Tukey *post-hoc* analysis showed that patients with a high disorganization score made more errors than controls (*p* < 0.01). The group effect did not reach significance level in the neutral condition, *F*_(2, 41)_ = 2.7, *p* = 0.076.

### Tap-sound asynchronies

**Figure 4:**
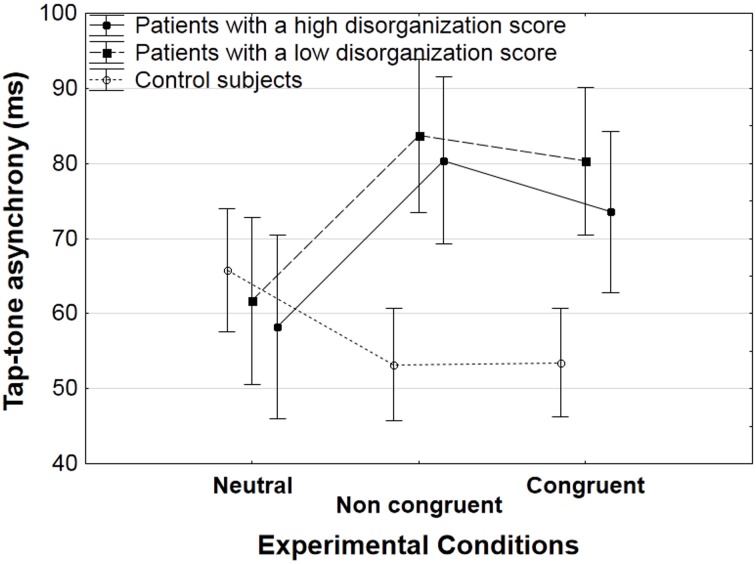
**Mean tap-tone asynchrony (with standard error of the means, SEM) on the 2d tap, for correct trials averaged across participants in each group (patients with a low and high level of disorganization, and controls) as a function of the experimental condition (neutral, non-congruent, congruent)**.

The recording of tap-sound asynchronies was aimed at checking to which extent participants benefited from the sound regularities and planned their tap in advance. Results on this parameter were identical in patients with a high and low score of disorganization, as illustrated in Figure 4 and thus, data was averaged across subgroups of patients.

A first analysis of variance conducted on tap-sound asynchronies showed a triple significant interaction between group (patients vs. controls), the tap order (first vs. second), and the experimental conditions (neutral, congruent, non-congruent), *F*_(2, 84)_ = 4.3, *p* < 0.05. In all participants, the tap-tone asynchrony was longer for the second than for the first tap, reflecting the rhythmic structure of the motor sequences. In patients, the tap-tone asynchrony increased by 41 ms between the first and the second tap, *F*_(1, 21)_ = 23.6, *p* < 0.001. In controls, it increased by a similar amount, i.e., by 41 ms, *F*_(1, 21)_ = 82, *p* < 0.001. In the following sections, we decompose the 3rd level interaction by distinguishing the first vs. second tap of a sequence of two.

#### First tap

For the first tap, tap-sound asynchronies were larger in patients than in controls [32 vs. 16 ms, *F*_(1, 42)_ = 5.7, *p* < 0.05]. However, this effect did not interact with the experimental conditions (*F* < 1).

#### Second tap

For the second tap, we observed an interaction between group and experimental conditions [*F*_(2, 84)_ = 10.3, *p* < 0.001). Decomposing this interaction showed opposite effects of the presence vs. absence of connecters in the patients and in the controls.

In controls, the tap-sound asynchronies were shorter in the presence of connecters than in the absence of connecters. This was reflected in a significant effect of experimental conditions [*F*_(2, 42)_ = 3.8, *p* < 0.05]. The effects were not strong enough to yield significant effects in the HSD Tukey *post-hoc* analysis, but sub-analyses showed that tap-sound asynchronies were shorter in the non-congruent than in the neutral condition [53 vs. 66 ms, *F*_(1, 21)_ = 4.4, *p* < 0.05], and that they tended to be shorter in the congruent than in the neutral condition [54 vs. 66 ms, *F*_(1, 21)_ = 4.2, *p* = 0.051].

These effects were reversed in patients. The tap-sound asynchronies were longer in the presence of connecters than in the absence of connecters. There was a significant effect of experimental conditions [*F*_(2, 42)_ = 6.6, *p* < 0.005], and the HSD Tukey *post-hoc* analysis showed that both the congruent and non-congruent conditions yielded longer tap-sound asynchronies than the neutral condition (77 ms in the congruent and 82 ms in the non-congruent conditions, vs. 60 ms in the neutral condition, *p* < 0.05 and *p* < 0.005, respectively).

It is to be noted that tap-sound asynchronies were no larger in the bad trials, i.e., in the inverted trials (data not shown).

### Correlations between performance and treatment

There were no significant correlations between performance and the dosage of neuroleptics (in chlorpromazine equivalent). In addition, the number of patients treated with typical neuroleptics was equivalent in both subgroups of patients (4 and 3). Finally, the dosage of neuroleptics did not differ significantly between the two subgroups of patients [*F*_(1, 20)_ = 1.3, *p* > 0.25]: 219 vs. 335 mg in the groups with the low and the high scores of disorganization, respectively. The non-significant differences in dosage were due to one patient having a higher dose than all others (1200 mg). The dosage was strictly identical between groups when this patient was excluded from the analyses: 219 vs. 239 mg. In the present paper, the subject was included in all analyses because when excluded result patterns were identical in all points. For example, the error rates remained abnormally high in the patient group for the non-congruent condition (30% without vs. 31.6% with this subject).

Correlations with clinical disorganization scores have been described above. There were no other significant correlations with clinical scores.

## Discussion

Our experimental design was aimed to explore the impact of visual grouping on the execution of a sequence of motor actions. In the present study, the participants' task was to produce pairs of taps on a touch screen in synchrony with alternated auditory rhythms. The results indicated that the connecters significantly impacted the planning and execution of the tap sequences both in controls and patients. More specifically, in healthy participants, there was a significant effect of the presence of connecters on tap-tone asynchrony, with a slight improvement in timing performances indexed through the reduction of tap-tone asynchronies. The amplitude of this effect was small in controls, but it contrasted with a large impairment in patients with schizophrenia. Indeed, not only the presence of connecters did not help but it was detrimental for all patients, whatever the degree of clinical disorganization. In addition, there was a specific difficulty in the non-congruent condition in those patients characterized by high scores of clinical disorganization. Overall, we suggest this easy non-verbal task may provide the means to gain a better understanding of the relationship between the disorganization symptoms and the use of cognitive representations for the planning and execution of structured motor behavior.

Several trivial explanations can be discarded. The results reported in the present study cannot be attributed to a generalized deficit in schizophrenia. Indeed, a generalized deficit results in amplified or reduced effects, as compared to those observed in controls because, e.g., patients are more sensitive to task difficulty than controls, irrespective of the mechanisms involved (Knight and Silverstein, 2001). A generalized deficit can hardly explain the opposite effects that we reported for tap-tone asynchronies in patients and in controls. It can neither predict the large congruency effects that we report in the disorganized patients, since such effects are in fact absent in the control group. A second point of importance is that we were able to eliminate an explanation in terms of neuroleptic treatment, inasmuch as both subgroups of patients had a similar treatment dosage and treatment type (typical vs. atypical) even though they differed markedly on performance profiles. Finally, the timing parameters that were computed confirmed that overall the patients were able to perform the task adequately: the tap-sound asynchronies were way below the latencies expected for simple reaction times (<200 ms), confirming that all three groups anticipated the timing of occurrence of the sounds to some extent. All participants used the auditory information to plan the sequential actions and thus, to perform in a predictive fashion following the auditory rhythm as instructed. This pattern of results was observed even in those patients who were characterized by high error rates (i.e., those trials produced with an inversed ratio), suggesting that difficulty was not in the motor task in itself.

Overall, these findings suggest that our paradigm may help to uncover a more selective impairment than a general planning/sequencing deficit. In the following, we decompose the different mechanisms involved in controls, and then suggest an interpretation of the impairments in relation to schizophrenia.

In the present study, because of the timing constraints, the task required participants to organize the different visual elements of action within pairs in order to prepare sequences of taps in a predictive manner. Tap-sound asynchronies indexed anticipation and planning, and tap-sound asynchronies for the second tap were shorter when connecters were present rather than absent. These effects suggest that connecters help controls to plan their sequence of taps more efficiently. This may be the case because under such conditions there is an organization correspondence between visual space and motor representations. This would be similar to congruency effects between action and sensory feedbacks, as described in the literature within the ideomotor theory (Prinz, 1987; Hommel et al., 2001; Shin et al., 2010). This would allow participants to know in advance where to expect connecters, in relation to the visual targets they need to point to. Even if this knowledge is not conscious, it would help participants to benefit from the congruency between expectation and true sensory feedback and thus, help to improve performance accuracy throughout the trial. Moreover, this might represent a possible mechanism allowing participants to avoid a deleterious effect of the non-congruent condition. It is indeed striking that control participants were as efficient in the congruent and in the non-congruent conditions, both for rates of errors and for tap-sound asynchronies. This is in marked contrast with what is observed in visual perception tasks for which a performance advantage is very commonly observed when the task is directed to a unique object (or group of objects) compared to that seen when the task is directed toward distinct, non-grouped objects (Duncan, 1984; Egly et al., 1994; Beck and Palmer, 2002; Palmer and Beck, 2007; van Assche et al., 2012). But it is the case that in the present task, participants benefited from the regularity of the organization to guide motor outputs. The most likely explanation is that healthy participants learned the correspondence between the tap pairs and the visual organization of the visual targets. In the non-congruent condition, this might have been facilitated by the fact that healthy participants are able to build a representation that binds unconnected targets together (Giersch and Rhein, 2008; van Assche et al., 2012). Inasmuch participants “re-grouped” unconnected targets, they quickly learned to expect an absence of connecters between those visual targets that were to be tapped together. This mechanism may have helped them overcome a possible motor-perceptual correspondence difficulty in the non-congruent condition. It would account for the advantage provided by connecters in the controls, and explain the lack of congruency effect.

In contrast to controls, the patients' tap-sound asynchronies increased when connecters were present. These results suggest that connecters further impaired the patients' ability to plan efficiently their tap sequence. It is the case that patients have been shown to be selectively affected in tasks requiring the anticipatory planning of sequences of actions (Jogems-Kosterman et al., 2001; Delevoye-Turrell et al., 2007; Grootens et al., 2009), and our results are consistent with this literature. Even though the tap-tone asynchronies were small and indicated that patients were planning their tapping sequence in a predictive manner up to a certain extent, asynchronies recorded in patients were much longer than those observed in controls in the presence of connecters. This suggests a difficulty in the patients to integrate the visual organization within the motor plan of the tap sequences. As a consequence patients did not benefit from the organization correspondence between visual information and motor representations. Moreover, if the visual organization is not integrated during the motor planning phase, visual information would then need to be processed during motor execution, which would increase furthermore the cognitive load of the motor task. This might have led patients to be more sensitive to the effects of congruency. The lack of congruency effects on tap-tone asynchrony certainly suggests that the conflict arising in the non-congruent condition affects motor execution but not motor planning.

The impact of the non-congruent condition is similar to those impairments previously reported in visual perceptual tasks (Giersch and Rhein, 2008; van Assche and Giersch, 2011; Giersch et al., 2012), which showed that patients have a difficulty with pairs of objects when these objects are not only individualized but belong to different groups. The present results suggest that this specific cognitive impairment in the perceptual domain directly impacts performance levels in the motor domain. The difficulty in the non-congruent condition is thus observed in those patients having a difficulty with unconnected figures belonging to different groups, i.e., patients with a high score of disorganization. It is possible that because patients do not integrate visual organization within their motor plan, they do not use connecters as landmarks to stabilize and/or improve their tapping accuracy. This would lead patients to switch more easily from the non-congruent to a congruent tapping mode, leading to an inversion of the alternated rhythm. It is this combination of planning and visual organization deficits that might account here for the large impairments reported in disorganized patients specifically in the non-congruent condition.

The present findings now need to be confirmed in larger groups of patients. However, our results suggest that the approach to combine a need to organize factors of different sensory modalities within a simple motor task may be a good lead in the development of more sensitive tools for the exploration of cognitive disorders and their relationship with clinical symptoms. It is noteworthy that the impairment on tap-tone asynchrony concerns all patients, contrary to the effect of the non-congruent condition on error rates. This suggests that the two variables tap into different mechanisms that contribute differently to clinical symptoms. As we have seen above, the lengthening of the tap-tone asynchrony suggests a difficulty in action planning, and this might be of significance at a clinical level. If patients cannot plan efficiently their actions, this means a loss of control on their behavior. Such a loss of control might pave the way for delusions of control, i.e., for the belief that one's own actions are controlled by an external force (Wilquin and Delevoye-Turrell, 2012). The impairment in planning described in our study might add up to those deficits already proposed to be involved in the emergence of such delusions (Frith, 2005). The effect of the non-congruent condition, on the other hand, appears to be rather related to known visual organization impairments. What remains to be seen, however, is to what extent the clear-cut performance differences observed between the two subgroups of patients correspond to true observable clinical differences. It is the case that clinical disorganization is evaluated on a continuum, whereas the present patterns of results suggest a subgroup division. Hence, further exploration is required to test the clinical validity of the subdivision that was proposed here.

Future studies will also reveal whether the present exploration complements existing measures of executive and motor performances related to disorganization in schizophrenia, e.g., neurological soft signs for motor control (Mohr et al., 1996; Tosato and Dazzan, 2005; Mechri et al., 2009) and the tower of London for executive functions (Greenwood et al., 2011). The tower of London requires participants to move discs from one peg to another by following a number of pre-determined rules. All these tasks require both motor coordination and motor planning. What our paradigm brings in addition is the possibility to distinguish planning from execution, and to isolate a condition that represents a peculiar difficulty for patients: regrouping visual items for fluent motor execution. This difficulty makes clinical sense inasmuch that it echoes the loosening of association originally described by Bleuler. Furthermore, it indicates that some associations are more difficult than others to performance for patients suffering from schizophrenia. In the present paradigm, associating items already bound together through automatic grouping mechanisms is not as difficult as associating items that are not only individualized but that belong to different groups of objects. This condition might be specifically difficult for disorganized patients.

This cognitive deficit for motor fluency might have been missed until now because pointing toward objects seems trivial. Indeed, in the present study, the control participants were very efficient in planning and executing rhythmic sequences of motor pointing actions toward unrelated spatial elements that belonged to different groups of objects. This ability is very useful in our multimedia daily experiences. Indeed, the visual environment is often crowded of distractors, and healthy individuals frequently plan pointing actions between objects, whatever their relative locations in space and in time (imagine tapping on the touchpad of your telephone). What usually seems so easy to us might, however, be much more difficult to perform for patients suffering from schizophrenia, and especially those patients characterized with high scores of clinical disorganization. What remains to be seen is whether the impairments observed here in a manual pointing task can also explain the patients' difficulties navigating visually through the visual environment, i.e., with ocular rather than manual movements. It has often been shown that patients have a reduced span of exploration when viewing a face or an abstract picture (Gaebel et al., 1987; Kojima et al., 1990; Gordon et al., 1992; Phillips and David, 1997; Loughland et al., 2002; Obayashi et al., 2003; Minassian et al., 2005; Delerue et al., 2010; Delerue and Boucart, 2012). Patients can miss part of a picture. It might be the case that impaired spontaneous exploration of natural scenes is due at least in part to a difficulty in planning and executing sequential eye movements between unrelated parts of a visual image.

### Conflict of interest statement

The authors declare that the research was conducted in the absence of any commercial or financial relationships that could be construed as a potential conflict of interest.
